# Thank you to reviewers

**DOI:** 10.1093/eurpub/ckae007

**Published:** 2024-02-05

**Authors:** Peter Allebeck, Diana Delnoij, Róza Ádány, Stefania Boccia, Alastair Leyland

A scientific journal exists only because of the good will and assistance of its reviewers. We can only publish approximately 25% of the papers we receive. In order to publish really high-quality papers, we depend on the expertise of many people. In 2023, a total of 406 reviewers provided their expert opinions for us to decide about the papers that we should publish. Their names are listed below, and we would like to take this opportunity to thank them.

Lucia Artazcoz, Spain

Mette Aadahl, Denmark

Adrian Aginagalde, United States

Utkarsh Agrawal, United Kingdom

Charles Agyemang, Netherlands

Linda Ahrenfeldt, Denmark

Eva Aizpurua, United States

Adebisi Akande, Canada

Yannis Alamanos, Greece

Tit Albreht, Slovenia

Denise Alexander, Ireland

Tamara Alhambra-Borrás, Spain

Mirjam Allik, United Kingdom

Bernt Alm, Sweden

Melody Almroth, Sweden

M. Amador, United States

Ridwanul Amin, Sweden

Lars Andersen, Denmark

Sven Andréasson, Sweden

Peter Angelopoulos, Greece

Thomas Anker, Denmark

Cigdem Apaydin Kaya, Turkey

Franklin Apfel, United Kingdom

Ramiz Arabaci, Turkey

Fatime Arenliu Qosaj, Albania

Nimmathota Arlappa, India

Bruno Arpino, United States

Anne-Charlotte BAS, France

Eva Bacsne Baba, Hungary

Mark Bakker, United States

Ileana Baldi, Italy

Birna Baldursdottir, Iceland

Tom Baranowski, United States

Angelo Barbato, Italy

Orna Baron-Epel, Israel

José Augusto Barreto Filho, Brazil

Pascal Bauer, United States

Flavia Beccia, Italy

Deepak Kumar Behera, Viet Nam

Ana Belmonte, Spain

Fernando Benavides, Spain

Merita Berisha, Albania

Lisa Berkman, United States

Sylvie Berruet, United States

Alison Bish, United States

Vesna Bjegovic Mikanovic, Serbia

Ingunn Bjornsdottir, Denmark

Jonas Björk, Sweden

Vendula Blaya-Nováková, Spain

Jenni Blomgren, Finland

Maristela Bohlke, Brazil

Alberto Borghetti, Italy

Anne Brabers, Netherlands

Maria Piedade Brandão, Portugal

Ruben Brondeel, Belgium

Laura Brunelli, Italy

Alessandra Buja, Italy

Alex Burdorf, Netherlands

Howard Burkom, United States

Andrea Buron, Spain

Hermann Burr, Germany

Henna Busk, Finland

Marcel Buster, Netherlands

Éva Bíró, Hungary

Daniela CHieffo, United States

Chiara Cadeddu, Italy

Eduardo Cadore,

Giovanna Elisa Calabrò, Italy

Martin Caraher, United Kingdom

Antonella Carcagni, United States

Margarida Cardoso, Portugal

R Casanas, Spain

Xavier Castells, United States

R Catalano, United States

Angela Chang,

Tracey Chantler, United States

Boris Chapoton, France

Elisabetta Chellini, Italy

Costas Christophi, Cyprus

Nain-Feng Chu, Taiwan

Miriam Colombo, United States

Leanne Coombe, United States

Martina Cornel, Netherlands

Yolima Cossio-Gil, Spain

Claudia Costa, Portugal

Claudio Costantino, Italy

Matty Crone, Netherlands

Paolo Crosignani, Italy

Joanne Csete, United States

Katarzyna Czabanowska, Netherlands

Lanfranco D'Elia, Italy

Shaonong Dang, China

Zuzana Dankulincova, Slovakia

Mike Daube, Australia

Daniela De Angelis, United Kingdom

Helene De Pauw, Belgium

Robby De Pauw, United States

Rosa De Vincenzo, United States

Ulrik Deding, Denmark

Evangelia Demou, United Kingdom

Daniel Dench, United States

Sarah Denford, United Kingdom

Jiawen Deng, United States

B Devleesschauwer,

Marcello Di Pumpo, Italy

Petra Dickmann, United Kingdom

Finn Diderichsen, Denmark

Maxim Dierckens, Belgium

Annette J Dobson, Australia

Felıcitas Domınguez-Berjon, Spain

Danny Dorling, United Kingdom of Great Britain and Northern Ireland

Anne Doyle, Ireland

Nico Dragano, Germany

Sven Drefahl, Sweden

Ruth Dundas, United Kingdom of Great Britain and Northern Ireland

Xavier Duran, Spain

Anna Dzielska, Poland

Frida Eek, Sweden

Ola Ekholm, Denmark

Marko Elovainio, Finland

Eric Emerson, United Kingdom

Petra Fadgyas-Freyler PhD, Hungary

John Fanourgiakis, Greece

Line Farah, United States

Sara Farina, Italy

Christina Fastl, United States

David Fauser, Germany

Albert Faye, France

Muhammad Fayyaz Nazir, United States

Bruno Federico, Italy

Miguel Felgueiras, Portugal

Joao Ferreira-Pinto, United States

Carmine Finelli, Italy

Claudia Fischer, Netherlands

Florian Fischer, Germany

Patricia Fitzpatrick, Ireland

Naomi Fleming, United States

Maria João Forjaz, Spain

Stefan Fors, Sweden

Rosemary Frasso, United States

Johan Fritzell, Sweden

Sara Fritzell, Sweden

Sari Fröjd, Finland

Judith Fuchs, Germany

Hugh Fulham-McQuillan, Ireland

Amélie Gabet, France

Carmelo Gabriele, Netherlands

Thomas Gamsjäger, Austria

Eva García-Álvarez, Austria

Jessica Gardner, United States

Lalit Garg, Malta

Elisabeth Garratt, United Kingdom

Natalia Gavrilova, United States

Karin Geffert, United States

Andrea Gentili, Italy

Laurent Gerbaud, France

Krisztina Gero, United States

Diana Giannarelli, United States

Alejandro Gil-Salmerón, United Kingdom

Judith Godin, Canada

Pere Godoy, Spain

Rosa Gofin, Israel

Virgilio Gomez-Rubio, United States

Pat Goodman, Ireland

Alexander W. Gorny, Singapore

Irina Grafova, United States

Mark Green, United Kingdom

Scott Greer, United States

Matthew Groenewold, United States

Enzo Grossi, United States

Jing Gu, United States

Maria Gualano, Italy

C Guijarro, United States

Alexandra Gyllenberg, Sweden

Juanita Haagsma, Netherlands

Luc Hagenaars, United States

Faisal Hakeem, United Kingdom

Kai Haldre, Estonia

Huiju Han, China

Dr Helen Harcombe, New Zealand

Anne Havermans, United States

Jenny Head, United Kingdom

Pontus Hedberg, United States

N Heinonen, United States

Kène Henkens, Netherlands

Gunnel Hensing, Sweden

Alina Herrmann, Germany

Marit Hinnosaar, United Kingdom

Anna-Clara Hollander, Sweden

Lihong Hu, United States

Jiayuan Huang, China

Juliette Hussey, Ireland

Margareeta Häkkinen, Finland

Ivo Iavicoli, United States

Greg Inglis, United States

Kaire Innos, Estonia

Paul Isenman, United States

Damir Ivankovic, Netherlands

Mikel Izquierdo, Spain

Marielle Jambroes, Netherlands

Domantas Jasilionis, Germany

Dmitri Jdanov, Germany

Junia Joffer, United States

Anders Johansson, United States

Heather Joshi, United Kingdom

Kerry Joyce, United Kingdom

Hendrik Juerges, Germany

Leena Kaila-Kangas, Finland

Ilya Kashnitsky, United States

Christina-Maria Kastorini, Greece

Lital Keinan-Boker, Israel

Viktorija Kesaite, United States

Malcolm King, Canada

Jonas Kinge, Norway

Maria Kippler, United States

Volodymyr Korolenko, Ukraine

Loes Kreeftenberg, Netherlands

Theocharis Kromydas, United Kingdom

Anna Krueger, United States

Anton Kunst, Netherlands

Ruth Kutalek, Austria

Karolina Kósa, Hungary

Sebastiano La Maestra, United States

Carlo La Vecchia, Italy

Mikko Laaksonen, Finland

Lies Lahousse, Belgium

Taavi Lai, Estonia

Patrizia Laurenti, Italy

Fernanda Leite, United States

Tinne Lernout, Belgium

Kate Lewis, United Kingdom

Alastair Leyland, United Kingdom

Jutta Lindert, Germany

Allan Linneberg, United States

Stefan Lipman, United States

Francesco Longo, Italy

Silvia Lopes, Portugal

Ulf Lundberg, Sweden

Raimundas Lunevicius, United Kingdom

Pål Martinussen, Norway

Johan Mackenbach, Netherlands

David Madden, Ireland

Kinsuk Maitra, United States

Sierk Marbus, Netherlands

Tanja Marschall, Germany

Homero Martínez, Canada

Kalle Matias Mattila, Finland

Mathew Mbwogge, United Kingdom

Martin McKee, United Kingdom

Philip McLoone, United Kingdom

Maria Melchior, France

Andriy Melnyk, Italy

Gregoire Mercier, France

Nicos Middleton, Cyprus

Alexander Miething, Sweden

Margherita Moretti, Italy

Hanns Moshammer, Austria

Antons Mozalevskis, Denmark

Massimo Mucciardi, Italy

J Murasko, United States

Mario Muselli, Italy

Mikko Myllykangas, United States

Julia Nakamura, United States

Tamsin Newlove-Delgado, United States

Paul Norman, United Kingdom

Mario Cesare Nurchis, Italy

Anna Odone, Italy

Beatrix Oroszi, Hungary

Tina Purnat, Sweden

Pia Pafundi, United States

Nikola Panic, United States

Matthew Parbst, Italy

Antoine Pariente, France

Timo Partonen, Finland

M. Isabel Pasarín, Spain

Tina Pasciuto, United States

Domenico Pascucci, Italy

Valeria Passos, United States

Roberta Pastorino, Italy

Jay Patel, United States

Julian Perelman, Portugal

Elena Petelos, Greece

Isabelle Peytremann-Bridevaux, United States

Angelo Maria Pezzullo, Italy

Sylvain Pichetti, France

Peter Piko, Hungary

Denise Pires Marafon, Italy

Sami Pirkola, Finland

Tom Platteau, Belgium

Hélène Poirel, Belgium

Johan Polderz Tiburg, United States

Anu Polvinen, Finland

Patrick Präg, United Kingdom

Rosa Puigpinós-Riera, Spain

Rui Qi, China

Jjiabi Qin, China

Amal Rahi, Lebanon

Justina Račaitė, Lithuania

Ettero Recchi, United States

Vivian Reckers, United States

Johan Rehnberg, Sweden

Sijmen Reijneveld, Netherlands

Grégoire Rey, France

Julien Riou, United States

Hannah Robertson, United States

Priscilla Robinson, United States

Vladimir Rogalewicz, Czech Republic

Dan Romer, United States

Aldo Rosano, Italy

Anna Laura Ross, United States

Alexandre Rossignol, United States

Rima Rouhana, Lebanon

Olivier Rubin, United States

Maria Sala, Spain

Eugenio Santoro, Italy

João Santos, Portugal

Rui Santos, United States

Ailiana Santosa, Sweden

Milena Santric- Milicevic, Serbia

Peter Sarich, United States

Romina Saucedo, United States

David Savitz, United States

Nicolò Scarsi, Italy

Lasse Scheel-Hincke, Denmark

Richard Shaw, United Kingdom

Wensong Shen, Hong Kong

Ilana Shoham-Vardi, Israel

Sven-Arne Silfverdal, Sweden

Zoran Simonovic, United States

Aïda Solé-Auró, Spain

Margit Sommersguter-Reichmann, Austria

Maria Specchia, Italy

Tanja Stamm, United States

Denes Stefler, United Kingdom

Tom Sterud, Norway

Tim Stockwell, Canada

Erwin Stolz, Austria

Saverio Stranges, Canada

Hitomi Suga, Japan

Derek Summerfield, United States

Reijo Sund, Finland

Magnus Svartengren, Sweden

Thomas Swanton, United States

Peter Söderlund, Finland

Jeppe Karl Sørensen, Denmark

Karl Tamussino, United States

Yelena Tarasenko, United States

Kyla Taylor, United States

Nicholas Taylor, Australia

Wendy Thompson, United States

Hazel Thornton, United Kingdom

Nuria Torner, Spain

Perihan Torun, Turkey

Ervin Toçi, Albania

Marta Trapero Bertran, Spain

Frank Trovato, Canada

Cleon Tsimbos, Greece

Kaitlyn Tsuruda, Norway

Angelica Valz Gris, Italy

Pierre Vanden Bussche, United States

Orsolya Varga, United States

Robert Verheij, Netherlands

Marieke Verschuuren, Netherlands

Gunnhild Vie, Norway

Leonardo Villani, Italy

Marianna Virtanen, Sweden

Pekka Virtanen, Finland

Melvin Vooren, United States

Vladimir Vukovic, Serbia

Seema Vyas, United States

Iris Wallenburg, Netherlands

David Walsh, United Kingdom

Jiunn Wang, United Kingdom

Jeremy Ward, France

Hajo Wildschut, Netherlands

Frans Willekens, Netherlands

Gregory Williams, United States

Gloria Wong, United States

Yo Ye, United States

Liyong Yuan, United States

Ángel R. Zapata-Moya, Spain

Marco Zappa, Italy

Virginia Zarulli, Denmark

Drieda Zaçe, Italy

Chunyu Zheng, United Kingdom

Pingping Zheng, China

Manhua Zhu, United States

Cheryl Zlotnick, Israel

Giulio Castelpietra, Italy

Mustafa Cetin, Turkey

Pauline de Heer, Netherlands

Chiara de Waure, Italy

Karine Gallopel-Morvan, France

Nóra Kovács, United States

Christel van Dijk, Netherlands

Jitse van Dijk, Netherlands

Alyson van Raalte, Germany

Maarten van Wijhe, Denmark

Ko van Wouwe, Netherlands

Sander van Zon, Netherlands

Monique van der Veen, Netherlands

J. van der Velden, Netherlands

Alberto Villani, Italy

Matthias von Herrath, United States

Per Olof Östergren, Sweden

Dorota Ćwiek, Poland

Tomaž Čakš, Slovenia

